# Analysis of Lens Thickness Distribution Based on Swept-Source Optical Coherence Tomography (SS-OCT)

**DOI:** 10.1155/2021/4717996

**Published:** 2021-12-30

**Authors:** Xi Feng, Yong Wang, Jianheng Liang, Yali Xu, Julio Ortega-Usobiaga, Danmin Cao

**Affiliations:** ^1^Aier School of Ophthalmology, Central South University, Changsha, Hunan Province, China; ^2^Aier Eye Hospital of Wuhan University, Aier Eye Hospital Group, Wuhan, Hubei Province, China; ^3^Department of Cataract and Refractive Surgery, Clínica Baviera-AIER Eye Hospital Group, Bilbao, Spain

## Abstract

**Objective:**

This study aimed to analyze the distribution of lens thickness (LT) and its associations in age-related cataract patients based on swept-source optical coherence tomography (SS-OCT).

**Methods:**

This cross-sectional study included 59,726 Chinese age-related cataract patients. Only right-eye data were included in the study. Repeated measures of ocular parameters were performed using an IOL Master 700 device. The distributions of ocular biometric data including anterior chamber depth (ACD), LT, axial length (AL), central corneal thickness (CCT), white-to-white (WTW), and mean keratometry (MK) and their associations with age were assessed. The anterior segment (AS) was measured as the sum of CCT, ACD, and LT, while the vitreous chamber depth (VCD) was calculated as the difference between AL and AS. The values of LT : AL, AS : AL, and VCD : AL in different AL groups and their changes are the main outcome measures used to observe the proportion of the anterior and posterior segments of the eye.

**Results:**

Biometric data were available for 59,726 individuals. The mean age was 68.81 years (range = 40–100); 40.62% were male and 59.38% were female. Mean anterior chamber depth (ACD) was 3.02 ± 0.44 mm, mean LT was 4.51 ± 0.44 mm, mean axial length (AL) was 23.89 ± 1.92 mm, mean central corneal thickness (CCT) was 0.53 ± 0.03 mm, mean white-to-white (WTW) was 11.64 ± 0.44 mm, and mean keratometry (MK) was 44.27 ± 1.65 diopter. Female patients had shorter AL, shallower ACD, smaller CCT and WTW, decreased LT, and steeper corneas (*p* < 0.005). ACD revealed the strongest negative correlation (*p* ≤ 0.001, *r* = –0.682) with LT. Age (*p* ≤ 0.001, *r* = 0.348) showed a moderate positive correlation, whereas MK (*p* ＜ 0.05, *r* = 0.011), CCT (*p* ≤ 0.001, *r* = 0.041) had a weak positive correlation and WTW (*p* ≤ 0.001, *r* = –0.034) had a weak negative correlation with LT. A nonlinear correlation was found between LT and AL. LT increased with age in both males and females. LT changed variably in eyes with AL less than 27 mm, LT decreased as AL increased, then LT gradually increased as AL increased in extremely long and extra-long eyes (*p* ≤ 0.001). LT : AL and AS : AL decreased as AL increased, VCD : AL gradually increased as AL increased in highly myopic eyes, and VCD : AL increased by about 0.01 for every 1 mm increase in AL.

**Conclusions:**

Among Chinese age-related cataract patients, we found LT to have the strongest relation with ACD. The lens was thicker in elderly patients and women. The correlation between LT and AL is not a simple negative correlation; with the increase of age, LT decreases first and then increases. The proportion of VCD is constantly rising with the elongation of AL.

## 1. Introduction

Globally, cataracts are the second leading cause of visual impairment and the leading cause of blindness. Song et al. [1] project that the number of people with age-related cataracts in China will rise to 187.26 million by 2050. In cataract surgery, new intraocular lens (IOL) power calculation formulas attempt to predict the effective lens position (ELP) by incorporating more individualized ocular parameters, such as lens thickness (LT) and white-to-white (WTW) [2]. The race and gender differences of ocular parameters are important to consider when optimizing earlier IOL power calculation formulas [3]. In this regard, the accuracy of biometric measurements is one of the main concerns in IOL power calculation, which is fundamental in treating cataract patients. The lens accounts for 20% of the eye's refractive power, and abnormal morphologic changes in the lens during accommodation are closely related to presbyopia [4]. Studies have shown that an increase in LT is a main cause of primary angle-closure glaucoma [5]. Lens parameters vary between races and ethnicities [6], and knowledge of their variations is crucial for understanding the pathogenesis, diagnosis, and management of ocular diseases.

Previous studies have assessed the distribution of anterior chamber depth (ACD), axial length (AL), and other ocular parameters in the European and black populations [7, 8]. In addition, many of the published studies were conducted using traditional optical biometry, with poor repeatability and accuracy for the measurement of LT in hard cataracts. Some used A-scan ultrasound biometry, which is limited by its high degree of dependence on the operator. A newer practice, swept-source optical coherence tomography (SS-OCT; IOL Master 700, Carl Zeiss-Meditec AG, Jena, Germany), has achieved superior rates of biometric data acquisition with its noncontact method and greater reproducibility and repeatability [9].

In this study, we included a large number of age-related cataract patients in China to ascertain the distribution of LT and its associations with age and other ocular parameters based on SS-OCT and to further investigate changes in LT and the anterior segment (AS) with the elongation of AL.

## 2. Methods

### 2.1. Patients

This study was conducted from January 2018 to December 2019, obtaining ocular biometric data from age-related cataract patients from the Aier Eye Hospital Group. The only inclusion criterion was an age of 40 or older. Exclusion criteria were ocular abnormalities, such as a history of ocular surgery (e.g., refractive surgery), corneal diseases, lens dislocations, active ocular inflammation, and ocular trauma. Only right-eye data were included. The need for informed consent was waived due to the retrospective nature of the study. The study was conducted in accordance with the principles of the Declaration of Helsinki and approved by the Aier Eye Hospital Group's ethics committee (2020IRBLW07).

### 2.2. Biometric Measurements

Gender and date of birth were recorded before biometric measurement. Ocular biometrics—measuring AL, ACD, LT, central corneal thickness (CCT), WTW, and mean keratometry (MK)—were performed using an IOL Master 700 SS-OCT optical biometer, which collects three measurements and provides the mean. All patients were tested by experienced examiners, and the examinations that yielded poor results (i.e., standard deviation (SD) ≥ 0.05 mm) in all parameters were excluded. LT values were compared for age and gender. To assess the correlation between LT and age, LT was stratified into 10-year age groups: 40–49 years, 50–59 years, 60–69 years, 70–79 years, and ≥80 years. A previous study found that other ocular parameters change largely with AL, suggesting the influence of AL on LT. Thus, the patients in this study were further divided into five groups according to their AL measurement: < 22 mm (short eyes), 22–24.99 mm (normal eyes), 25–26.99 mm (moderately long eyes), 27–29.99 mm (long eyes), and ≥30 mm (extra-long eyes). AS was obtained as the sum of CCT, ACD, and LT [10]. Vitreous chamber depth (VCD) is the distance between the posterior lens capsule and the retinal pigment epithelium. Since VCD is not measured directly with the device, ACD, LT, and CCT (mm) values were deducted from AL to calculate VCD. The LT to AL ratio (LT : AL), AS to AL ratio (AS : AL), and VCD to AL ratio (VCD : AL) were measured using SPSS, version 22.0 (IBM, USA).

### 2.3. Statistics

Statistical analysis was performed using SPSS, version 22.0 (IBM, USA). The normality of the data was checked with a Kolmogorov–Smirnov (K–S) test. Each ocular biometric index was described as the mean and standard deviation. Spearman's correlation coefficients and scatterplots were used to demonstrate correlations between LT and other ocular parameters. We then performed backward stepwise multivariate regression analyses with LT as the dependent parameter. As independent parameters, we used age or ocular parameters that were significantly associated with LT in the univariate analyses. Independent sample *t*-tests were used to analyze differences between the values obtained from the data. One-way ANOVA was used to analyze differences in the five AL subgroups. All tests of association were considered statistically significant at *p* < 0.05.

## 3. Results

### 3.1. Characteristics

**Figure 1:**
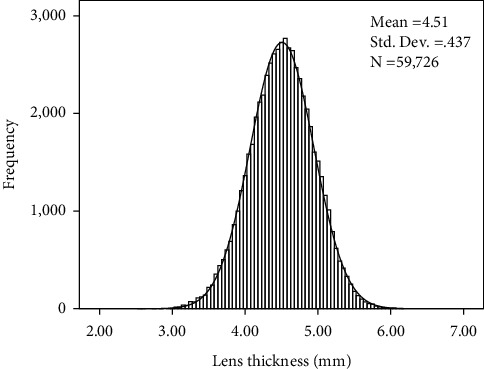
Histogram showing the distribution of lens thickness in Chinese cataract patients.

A total of 59,726 participants were included in this study. Their ages ranged from 40 to 100 years, with a mean age of 68.81 years. Females comprised 35,464 participants (59.38%), and 24,262 (40.62%) were males. Mean ACD was 3.02 ± 0.44 mm, mean AL was 23.89 ± 1.92 mm, mean CCT was 0.53 ± 0.03 mm, mean WTW was 11.64 ± 0.44 mm, and MK was 44.27 ± 1.65 diopter (D).  Figure 1 shows the frequency distribution of LT among the study population, with a mean value of 4.51 ± 0.44 mm.

### 3.2. Comparisons of Lens Thickness and Other Ocular Parameters, Stratified by Gender and Age

Lens thickness stratified by gender and age is shown in Table 1. When stratified by gender, there was a statistically significant difference in LT between males and females in every age group, except in the 40–49 group (*p* = 0.100) and the ≥80 group (*p* = 0.660). Females had lower mean AL, ACD, LT, CCT, and WTW, and flatter MK than males (all *p* ≤ 0.001). In addition, when LT was stratified by age in either men or women, LT increased gradually with age (for all age groups, *p* < 0.005). LT increased by about 0.014 mm per year in both males and females.

### 3.3. Associations between Lens Thickness, Age, and Other Parameters of the Anterior Segment

**Figure 2:**
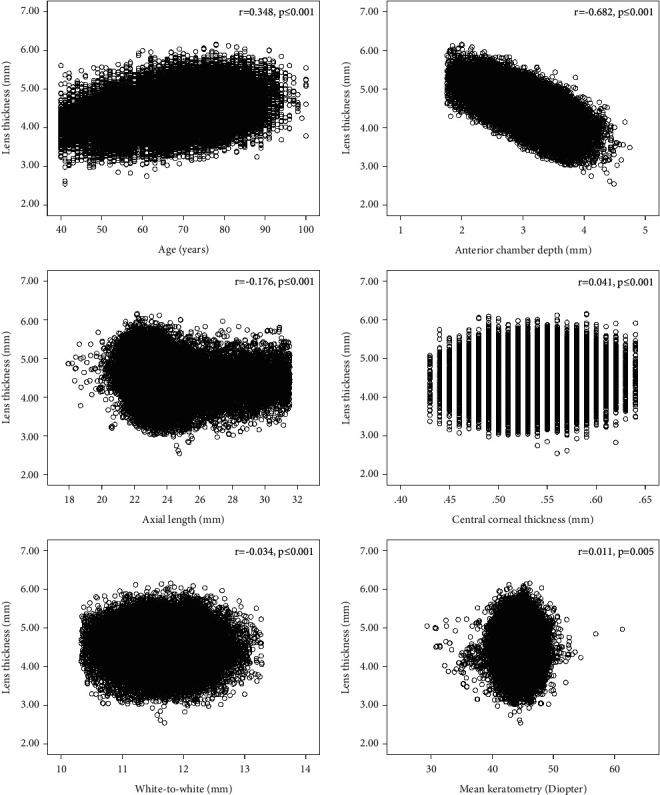
Correlations of lens thickness with age and other ocular biometric parameters.

The scatterplots for LT and age, ACD, AL, WTW, CCT, and MK are shown in Figure 2. Spearman's correlation revealed that LT was positively correlated with age (*p* ≤ 0.001 and *r* = 0.348) and negatively correlated with ACD (*p* ≤ 0.001 and *r* = –0.682). A nonlinear correlation was found between LT and AL, whereas CCT (*p* ≤ 0.001 and *r* = 0.041), WTW (*p* ≤ 0.001 and *r* = –0.034), and MK (*p* ≤ 0.05 and *r* = 0.011) were weakly correlated with LT (Table 2). A multivariate analysis was performed, which included all parameters that were significantly associated with the dependent variable of LT in the univariate analysis. This analysis revealed that LT was associated with older age (*p* < 0.001 and *β* = 0.186), longer AL (*p* < 0.001 and *β* = 0.216), and deeper ACD (*p* < 0.001 and *β* = 0.729; model adjusted *R*^2^ = 0.565).

### 3.4. Analysis of Lens Thickness, Stratified by Axial Length

The numbers and percentages of short eyes (<22 mm), normal eyes (22–24.99 mm), long eyes (25–26.99 mm), extremely long eyes (27–29.99 mm), and extra-long eyes (≥30 mm) were 3807 (6.4%), 46,269 (77.5%), 4544 (7.6%), 3761 (6.3%), and 1345 (2.3%). The scatterplot shown in Figure 2 indicates that LT and AL were not linearly correlated. After adjusting for age and gender, LT was greatest (4.68 mm) in the short eyes group and smallest (4.34 mm) in the moderately long eyes group. In short, normal, and moderately long eyes, LT decreased as AL increased, but in long and extra-long eyes, LT gradually increased as AL increased. This difference was statistically significant (*F* = 664.079 and *p* < 0.001).

**Figure 3:**
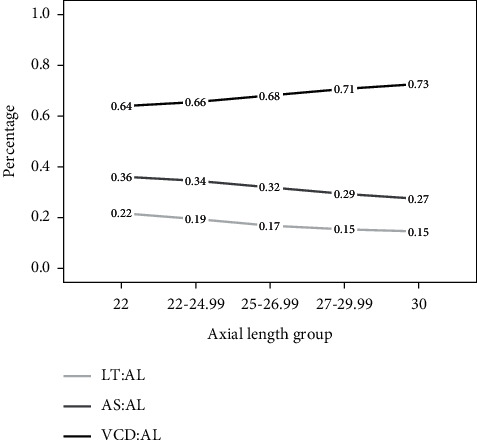
Proportions of lens thickness, anterior segment, and vitreous chamber depth stratified by axial length in Chinese cataract patients.

In addition, we calculated the values of LT : AL, AS : AL, and VCD : AL for all five AL groups.  Figure 3 shows the proportions of LT, AS, and VCD stratified by AL. LT : AL and AS : AL decreased when AL increased, and VCD : AL gradually increased when AL increased in highly myopic eyes. This difference was found to have very high statistical significance (*p* < 0.001). VCD : AL increased by about 0.01 for every 1 mm increase in AL.

## 4. Discussion

In refractive cataract surgery, there are many benefits of IOL power calculation based on accurate ocular biometry. SS-OCT has enabled two-dimensional tomographic visualization of ocular structures in an acquisition process faster than other optical biometers can achieve, such as partial coherence interferometry devices. One review indicated that SS-OCT-based ocular biometers have achieved accurate and repeatable outcomes, and such biometers are likely to become the gold standard of ocular biometry [11].

In our study of 59,726 cataract patients in China, the mean LT was 4.51 ± 0.44 mm. This is greater than the LT of a Portuguese cataract sample in a previous study (mean age = 69 ± 10 years; method: optical low-coherence reflectometry; LT = 4.32 ± 0.49 mm) [8] and the LT of an American cataract sample (mean age = 74.37 ± 8.93 years; method: A-scan ultrasound; LT = 4.93 ± 0.56 mm) [12], but it is smaller than the LT of 78 Japanese cataract patients (mean age = 72.1 ± 8.1 years; method: deep-range swept-source anterior segment optical coherence tomography; LT = 4.56 ± 0.39 mm) [13]. These differences could stem from the diverse age and racial groups included in the studies' samples.

Furthermore, we found that females have significantly different ocular biometrics than males. On average, females have shorter AL (mean = 23.76 mm versus 24.08 mm and *p* ≤ 0.001), shallower ACD (mean = 2.96 mm versus 3.12 mm and *p* ≤ 0.001), decreased LT (mean = 4.50 mm versus 4.52 mm and *p* ≤ 0.001), smaller CCT (mean = 0.53 mm versus 0.54 mm and *p* ≤ 0.001), shorter WTW (mean = 11.55 mm versus 11.78 mm and *p* ≤ 0.001), and steeper MK (mean = 44.61 mm versus 43.76 mm and *p* ≤ 0.001). We found that male cataract patients had greater LT than females, which contradicts the results of He et al., who found that LT tended to be greater in women [14]. The reasons for this discrepancy between the studies remain unclear. Hoffer and Savani compared mean LT between different races and genders and found no statistically significant differences [15]. Studies have confirmed that females have more crowded AS structures. Also, females, older age groups, and those with shorter AL, shallower ACD, and greater LT are at risk of angle closure [5, 16]. To better explain the role that lens parameters have in the pathogenesis of primary angle-closure glaucoma, Nongpiur et al. [17] included lens vault (LV) as a risk factor for angle closure (OR = 48.1). However, LT had no obvious correlation with angle closure (OR = 1.78 and 95% CI [0.76, 4.16]). In this previous study, females had decreased LT compared with males, which suggests that, in addition to LT, changes in the position and morphology of the lens in the anterior chamber increase the incidence of glaucoma in females. This view was subsequently reported in a Singaporean study [18].

In our study, greater LT is associated with increasing age, which corroborates previous studies; LT increased due to the continuous production of newly produced lens fibers in the equatorial region of the lens [19]. In addition, the aging of the lens is associated with a loss of elasticity; the lens gradually becomes rigid and loses its ability to accommodate, which further increases the anterior-posterior diameter of the lens [20]. This age-related growth of the lens plays a major part in the mechanisms leading to primary angle-closure glaucoma. Therefore, cataract extraction combined with intraocular lens implantation has a beneficial effect on older patients with primary angle-closure glaucoma, which replace a thick crystalline lens with an intraocular lens of less than 1.0 mm, thereby deepening the anterior chamber and alleviating pupillary block. A previous study also found that lens extraction showed great efficacy and safety and was more cost-effective than laser peripheral iridotomy [21].

We found that, in the groups with short, normal, and moderately long eyes, LT decreased as AL increased, but it gradually increased in the groups with long and extra-long eyes (*p* ≤ 0.001). Previous studies have revealed that LT is negatively associated with ocular AL [22, 23]. However, the emmetropization process is dynamic and continuous throughout the life span [24]. All ocular parameters in the anterior and posterior segments change with the growth of the eye. A simple analysis of the correlation between LT and AL cannot fully reflect the changes in AS or VCD vis-à-vis AL because the growth of the eye is highly disproportionate. Contrary to previous studies, our study found that the ratio between LT and AL is not entirely linear. When AL was less than 27 mm, LT gradually decreased as AL increased, while in eyes where AL was greater than 27 mm, LT gradually increased as AL increased. The impact of LT on IOL calculation has been confirmed in a previous study, which stated that formulas for the myopic shift with thin lenses and formulas for the hyperopic shift with thick lenses, especially in Haigis, Hill-RBF, version 2.0 [25]. As LT begins to increase as AL increases above 27 mm, some predictive error may enter into IOL power calculation and ELP for these highly myopic eyes. Our findings reveal that the ratios of LT : AL and AS : AL decreased with increased AL, but VCD : AL increased. This suggests that the posterior segment of the eye increased with the increase in AL. Takkar's study showed that the posterior segment seems to make up a greater proportion of the eye length as the AL increases [10]. Compared with a normal or hyperopic eye, a larger proportion of a myopic eye is occupied by the vitreous chamber. Patients with severe myopia can experience a series of degenerative fundus complications, such as macular degeneration and retinal detachment [26]. The changes in posterior chamber depth in highly myopic patients should be used as an indicator of related fundus complications, reminding us that doctors should increase fundus follow-up frequency, and posterior sclera reinforcement can be taken into consideration to delay the continuous extension of the axials and improving visual function when necessary.

Apart from its retrospective nature, this study has a significant limitation: LT varies with different types of cataracts [23]. No associations between LT and different types of cataracts were considered, and this may have affected the LT data.

In summary, our study described the distribution of LT by age and gender in Chinese cataract patients based on SS-OCT, revealing the most important determinants of LT : LT increased with age, both in females and males; the correlation between LT and AL was not simply negative, but in long eyes, LT increased with the elongation of AL. The proportion of LT and AS of the eye decreased with longer ALs compared with VCD constantly rising. This suggests that we should consider changes in posterior chamber depth in myopic patients.

## Figures and Tables

**Table 1:** Lens thickness in Chinese cataract patients, stratified by gender and age.

Age group	Male		Female		*p* value
	*N*	Mean ± SD (mm)	*N*	Mean ± SD (mm)	
40–49^a^	1594	4.14 ± 0.01	1995	4.14 ± 0.01	0.100
50–59	3157	4.31 ± 0.01	4202	4.28 ± 0.01	＜0.005
60–69	7200	4.50 ± 0.01	11258	4.46 ± 0.01	＜0.005
70–79	8441	4.60 ± 0.01	12609	4.58 ± 0.01	＜0.005
≥80	3870	4.70 ± 0.01	5400	4.69 ± 0.01	0.660
ALL^b^	24262	4.52 ± 0.00	35464	4.50 ± 0.00	＜0.005

SD: standard deviation. ^a^ Mann–Whitney *U* test. ^b^ Analysis of covariance after adjusting for age.

**Table 2:** Associations (Pearson's correlation) between lens thickness and other ocular parameters in Chinese cataract patients.

Parameters	Age (years)	ACD (mm)	AL (mm)	MK (D)	WTW (mm)	CCT (mm)
*R*	0.348	−0.682	−0.176	0.011	−0.034	0.041
*p*	≤0.001	≤0.001	≤0.001	0.005	≤0.001	≤0.001

## Data Availability

The data used to support the findings of this study are available from the corresponding author upon reasonable request.
